# Influence of Stretching on Liquid Transport in Knitted Fabrics

**DOI:** 10.3390/ma16052126

**Published:** 2023-03-06

**Authors:** Małgorzata Matusiak, Otgonsuren Sukhbat

**Affiliations:** Faculty of Material Technologies and Textile Design, Institute of Architecture of Textiles, Lodz University of Technology, 90-924 Lodz, Poland

**Keywords:** knitted fabrics, comfort, moisture, stretching, liquid, absorption, spreading

## Abstract

The transport of liquid sweat in clothing worn close to human skin is very important from the point of view of the thermo-physiological comfort of clothing users. It ensures the drainage of sweat secreted by the human body and condensed on the human skin. In the presented work, knitted fabrics made of cotton and cotton blends with other fibers (elastane, viscose, polyester) were measured in the range of liquid moisture transport using the Moisture Management Tester MMT M290. The fabrics were measured in unstretched form and stretched to 15%. Stretching of the fabrics was performed using the MMT Stretch Fabric Fixture. Obtained results confirmed that stretching significantly changed the values of parameters characterizing the liquid moisture transport in the fabrics. Before stretching, the best liquid sweat transport performance was stated for the KF5 knitted fabric made of 54% cotton and 46% polyester. For this, the greatest value (10 mm) of maximum wetted radius for the bottom surface was obtained. The Overall Moisture Management Capacity (OMMC) of the KF5 fabric was 0.76. This was the highest value among all values obtained for the unstretched fabrics. The lowest value of the OMMC parameter (0.18) was stated for the KF3 knitted fabric. After stretching, the KF4 fabric variant was assessed as the best one. Its OMMC improved from 0.71 before stretching to 0.80 after stretching. The value of the OMMC for the KF5 fabric remained after stretching at the same level (0.77) than before stretching. The most significant improvement was observed for the KF2 fabric. Before stretching, the value of the OMMC parameter for the KF2 fabric was 0.27. After stretching, the OMMC value increased to 0.72. It was also stated that the changes in the liquid moisture transport performance of the investigated knitted fabrics were different for the particular fabrics being investigated. Generally, in all cases, the ability of the investigated knitted fabrics to transfer liquid sweat was improved after stretching.

## 1. Introduction

As has been proven in many studies [1,2,3,4], clothing worn close to the body—underwear or others, such as T-shirts and sportswear—has a significant impact on the user’s thermal sensations. During hard exercise and other intensive activity, the sweat produced by the human body should be transferred through the clothing material assembly to the ambient air. If this is not the case, the moisture accumulates in the clothing. 

Underwear usually has been made of knitted fabrics in the form of T-shirts and shorts. Underwear, as the body’s “second skin”, has the function of ensuring the physiological comfort of the human body. Due to this fact, the choice of material for underwear is particularly important. Thus far, in the underwear market, cotton and other cellulose fibers (viscose) have been the main raw materials applied in underwear and clothing worn next to the skin. However, underwear made of cellulose fibers has strong hygroscopicity and a weak dehydration ability (difficult to dry). It is a common, traditional opinion that cotton or cotton-dominated fiber blends are the best choice for everyday underwear. However, the cotton fibers absorb sweat in the liquid form and become wet. At the same time, cotton fabric sticks to the user’s skin and becomes unpleasant to the touch, causing discomfort.

Everyday underwear material must be able to allow air to pass through and absorb perspiration without interfering with the body’s thermal conductivity in hot weather, and it must function in combination with other layers of clothing to maintain the body’s thermal balance in cold weather. Knitted fabrics are used in designing active clothing such as sportswear [4,5,6,7,8]. In recent years, in sportswear, innovative, advanced fibers, yarns and fabrics have been applied, such as profiled fibers (Coolmax), ThermoCool, TransDry, Wicking Windows, etc. They are especially designed to improve moisture management in sportswear and at the same time to influence in a positive way the wearer’s effectivity [6,9]. 

Many efforts are made to improve the quality and performance of textiles and clothing. Especially in the case of fabrics for underwear, the comfort-related properties of textile materials attract the attention of scientists. However, in the majority, the investigations are aimed at the water vapor permeability of the fabrics [10,11,12]. Water vapor permeability concerns moisture transmission in the form of gas. This property is especially important as higher levels of activity and/or climatic conditions cause intensive sweating and the sweat must be rapidly managed by the clothing. When the water vapor permeability is too low, the produced sweat condenses on the human skin. In this case, it is necessary to ensure the transport of moisture in the form of liquid. Thus, from the physiological comfort point of view, it is also necessary to characterize the materials in the aspect of the transfer of sweat in the form of liquid [13].

Measurement of liquid moisture transport can be performed by using the Moisture Management Tester by SDL Atlas Ltd. (Rock Hill, SC, USA). The device was developed especially for the assessment of textile materials in the aspect of their ability to transfer liquid moisture. The instrument measures the dynamic liquid transport in textiles in three aspects [14,15,16,17,18]:

- absorption of liquid moisture by the inner and outer surfaces of the fabric;
- spreading of the liquid on both (inner and outer) surfaces;
- one-way transfer of liquid moisture from the inner surface to the outer surface of the fabric.

In the case of clothing worn close to human skin, such as sportswear, underwear, T-shirts, etc., as was mentioned earlier, it is usually made of knitted fabrics. Knitted fabrics are stretchable. For usage in underwear and other clothing worn close to human skin, the fabric is in the stretched form. Stretching causes changes in the fabric structure, especially its compactness. As a result, some properties of the fabrics are also changed. Stretching influences the ability of fabrics to transfer liquid moisture. Stretching changes the sizes of pores in fabrics. The pores become larger. At the same time, the capillaries between fibers and yarns in the fabric structure are changed. This changes the conditions of liquid transport in fabric. Taking this into account, in many cases, the characterization of liquid transport in knitted fabrics in the relaxed (unstretched) form is not sufficient to determine the ability of the fabrics to ensure physiological comfort, especially in the context of draining liquid sweat from the skin of the wearer of the garment. 

SDL Atlas developed a small device—the MMT Stretch Fabric Fixture [19,20,21]—which makes it possible to prepare stretched samples for the measurement of liquid moisture transport in fabrics. The device is an appendix to the Moisture Management Tester. It enables the stretching of fabrics from 15% to 50% in 5% increments. 

The liquid moisture transport in knitted fabrics has already been the object of investigations [22,23,24]. However, the works published until now present only the results of liquid moisture transport in fabrics in a relaxed (unstretched) state. Suganthi et al. investigated the influence of the raw material composition and structure of bi-layer knitted fabrics on the liquid transport properties [22]. They stated that all the investigated structural parameters (yarn combinations in the inner layer and outer layer, yarn count, fabric thickness, stitch density and mass per unit area) influences the parameters provided by the MMT. Chen et al. investigated weft-knitted fabrics in plating structures made of cotton, polypropylene and polyester with different numbers of filaments [23]. The fabrics were tested by a moisture management tester, vertical wicking and drying test. It was found that the large water absorption difference and the large filament density difference between the inner and outer side of the fabric could result in better water absorption and drying ability. Kumar et al. also investigated layered knitted fabrics in the context of their ability to transfer liquid moisture. They investigated eri silk/micro-denier polyester/bamboo and eri silk/micro-denier polyester/Tencel-plated interlock fabrics [24]. The authors confirmed that the plated interlock knitted structure highly influenced the moisture management properties. When compared to the eri silk/micro-denier polyester/bamboo and Tencel combination, excellent moisture management properties were observed in the eri silk/micro-denier polyester/Tencel combination plated interlock knitted fabrics. 

All mentioned investigations have been performed for knitted fabrics in the relaxed (unstretched) form. The scientists analyzed the influence of the structural parameters on the liquid transport in knitted fabrics. As we know, the structure of the knitted fabrics can be changed according to needs or application an unlimited number of times. Each change in the structure leads to a change in the liquid transport properties. Until now, only a few works have been published concerning fabrics in the stretched state with a precisely defined stretch percentage [20,21]. They have been published by the authors of the current work. In our first publication [20], we presented preliminary trials of the application of the MMT Stretch Fabric Fixture in the preparation of stretched samples for measurement by means of the MMT. The publication was based on one variant of knitted fabric for babies’ clothing and different stretch percentages. This work allowed us to recognize the advantages and limitations of the MMT Stretch Fabric Fixture. In our next publication, we showed the influence of stretching on the moisture management properties of different variants of cotton and cotton blend knitted fabrics [22]. In this publication, the results are presented in a short manner, without an in-depth explanation of the results. The MMT Stretch Fabric Fixture has been also described only in our works. The description was rather general, without details. This is a new device that extends the research possibilities of textile materials, especially stretchable ones. However, it is still not recognized widely. 

The aim of the presented work was to investigate the influence of stretching on the parameters characterizing the liquid moisture transport in cotton and cotton blend knitted fabrics. Five commercially available T-shirts made of knitted fabrics were the objects of the investigations. The fabrics applied in the T-shirts were measured in terms of their ability to transfer liquid moisture. Measurement was performed by means of the Moisture Management Tester M290. Stretching the fabrics was achieved using the MMT Stretch Fabric Fixture. The obtained results allowed us to assess the influence of stretching on the liquid transport characteristics of the investigated knitted fabrics. An additional aim of our work was to provide information about the MMT Stretch Fabric Fixture as a new device, not recognized until now but very useful in textile materials’ assessment. In the current work, we also explain possible reasons for the great scattering of the results from the MMT.

## 2. Materials and Methods

Five types of knitted fabrics applied in commercially available T-shirts were the objects of the investigations. They were made of cotton, cotton with 3% elastane, cotton with 5% viscose and a 54% cotton/46% polyester blend. The basic structural parameters of the investigated fabrics are presented in Table 1. 

**Figure 1:**
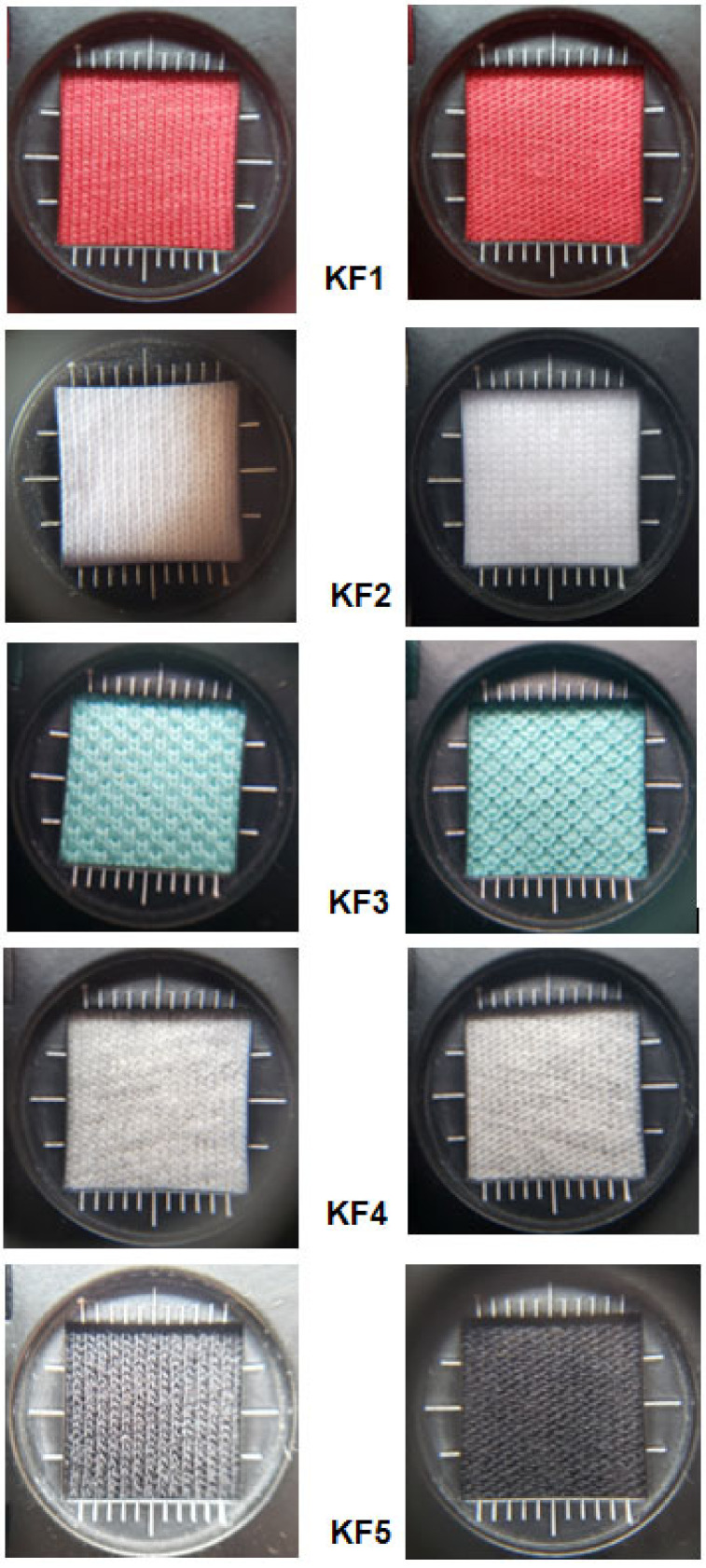
Images of the investigated knitted fabrics.

Figure 1 presents the right (bottom/outer) and left (top/inner) sides of the investigated fabrics. The images were obtained using the weaving magnitude in order to show the structure of both surfaces—the inner and outer parts of the investigated fabrics. In the figure, on the left, the right side of the fabric is presented; on the right, the left surface of the fabric is shown. 

Measurement was performed by means of the Moisture Management Tester (SDL Atlas, Rock Hill, SC, USA) according to the procedure described in the AATCC Test Method 195-2017 [15]. The MMT is controlled by a PC and the MMT290 software (Rock Hill, SC, USA). For measurement, square samples with dimensions of 80 mm × 80 mm were cut from the measured fabric. For each fabric tested, 5 repetitions of measurement were performed. While measuring, a pre-defined amount of testing solution (especially prepared synthetic sweat) was introduced onto the upper side (top, skin side) of the fabric in the first 20 s of measurement, and then the testing solution was transferred onto the material in three directions [14,15]:

- spreading on the upper surface of the sample;
- transferring through the fabric from the upper surface to the bottom surface;
- spreading on the bottom surface of the sample.

Depending on the hygroscopicity of the investigated fabric, some amount of liquid can be absorbed by the fibrous material of both surfaces. 

Measurement was performed in standard climatic conditions: 20 ± 2 °C temperature and 65 ± 5% relative humidity. The values of the following parameters were determined:

- WTT—wetting time of top surface, in [s];
- WTB—wetting time of bottom surface, in [s];
- TAR—absorption rate of top surface, in [%/s];
- BAR—absorption rate of bottom surface, in [%/s];
- MWRT—maximum wetted radius for top surface, in [mm];
- MWRB—maximum wetted radius for bottom surface, in [mm];
- SST—spreading speed on top surface, in [mm/s];
- SSB—spreading speed on bottom surface, in [mm/s];
- R—accumulative one-way transport index, [−];
- OMMC—Overall Moisture Management Capability, [−].

**Figure 2:**
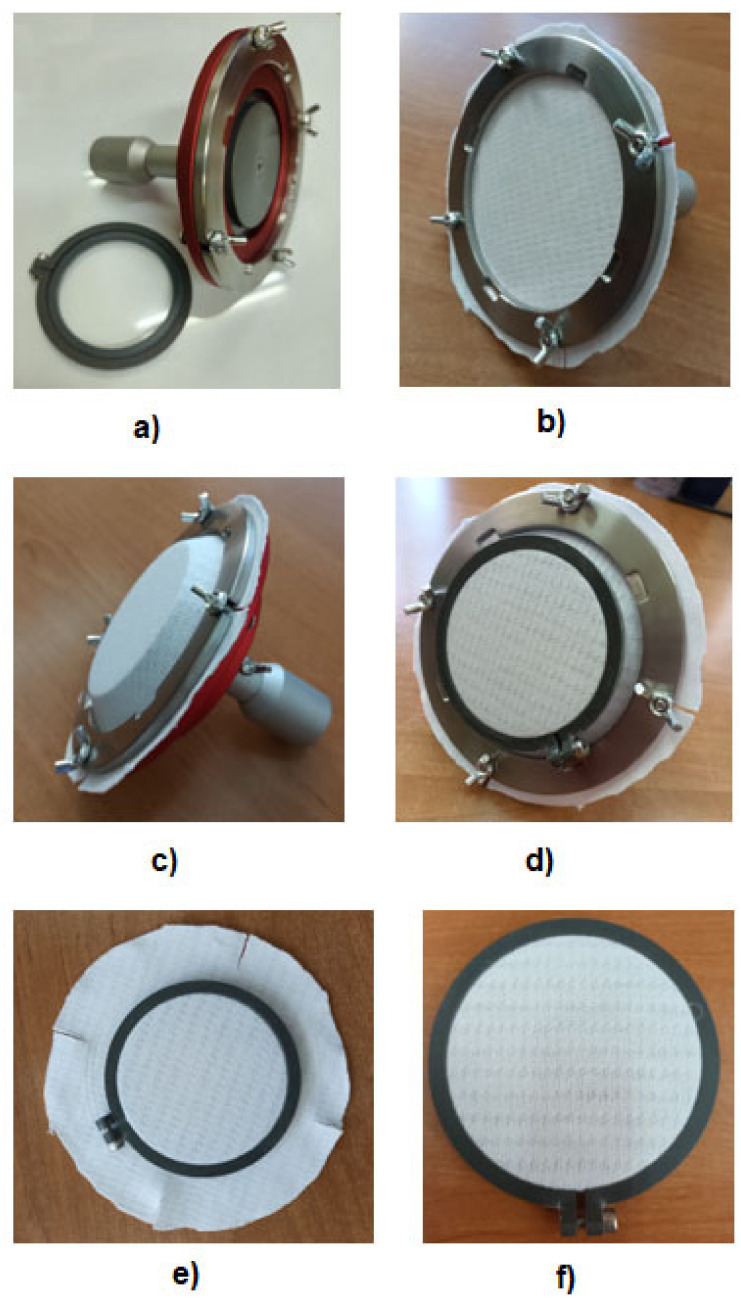
Stretching the fabrics by means of the MMT Stretch Fabric Fixture: (**a**) SFF device, (**b**) sample placement, (**c**) sample stretched, (**d**) locking the sample, (**e**) sample removed from the SSF, (**f**) stretched sample ready for measurement.

The MMT Stretch Fabric Fixture (SDL Atlas, Rock Hill, SC, USA) (Figure 2a) was applied to precisely stretch the fabrics. The device can extend the fabric to different sizes by up to 50%. However, our preliminary investigations showed that it is difficult to reach more than 30% for standard clothing materials. Additionally, thick materials such as polar ones cannot be stretched using the MMT Stretch Fabric Fixture because they are too thick to be locked in the clamp. The question is how to stretch standard woven fabrics without elastomeric yarns. This will be the focus of our future investigations. 

In the presented investigations, the knitted fabrics were stretched to 15%. According to the device instruction manual [19], the procedure of stretching was the following:

- placement of a round sample of 140 mm diameter on the table of the device and holding it by the fabric weight (Figure 2b);
- stretching the sample to the precisely determined stretch using the device insert (Figure 2c);
- locking the stretched sample in the fabric clamp (Figure 2d);
- removal of the stretched sample from the device (Figure 2e);
- trimming the excess fabric beyond the clamp circumference to obtain the specimen ready to be measured (Figure 2f).

**Figure 3:**
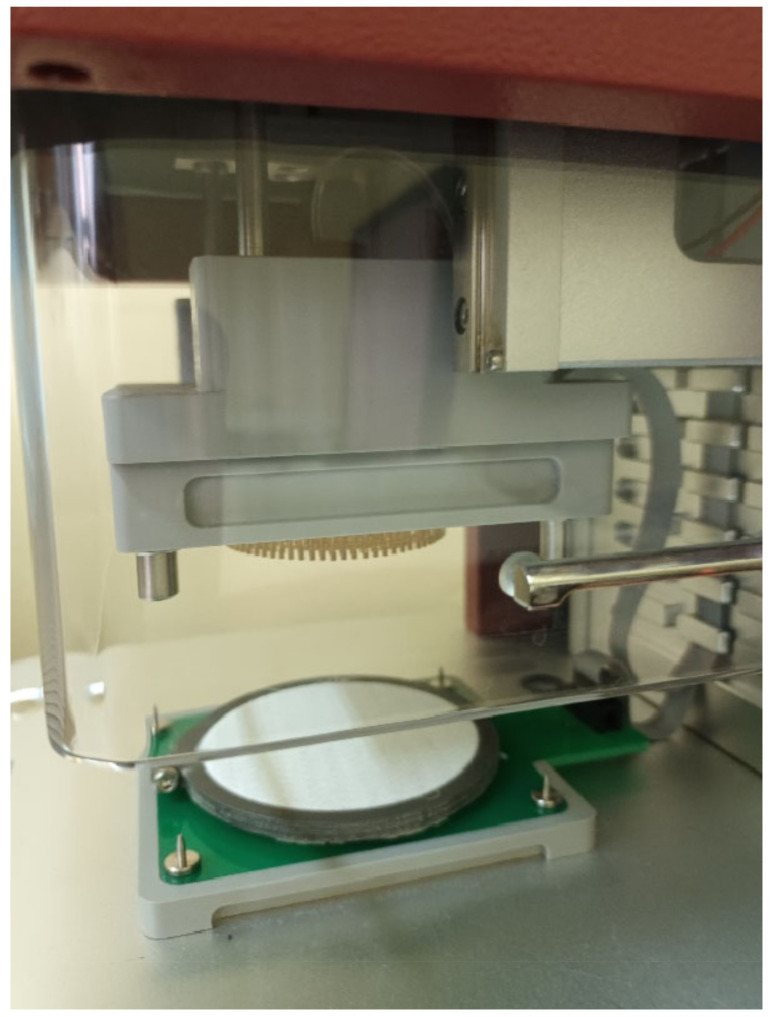
Placement of the stretched sample in the MMT.

The sample prepared in such a way was placed in the M290 MMT test area (Figure 3).

Statistical analysis of the results was performed using the multi-factor analysis of variance (ANOVA) available in TIBCO STATISTICA version 13.3 (TIBCO Software Inc., Palo Alto, CA, USA).

## 3. Results and Discussion

The results from the MMT for the knitted fabrics in the relaxed (unstretched) state are presented in Table 2 and Table 3. The tables present the mean values of particular parameters determined by the MMT and the standard deviation of results in brackets. 

On the basis of the results for the unstretched fabrics, it was stated that the investigated fabrics differed in terms of all parameters characterizing the liquid moisture transport through them. We also observed great variation in the results. 

The wetting time (WTT, WTB) denotes the time at which the top and bottom surfaces of the measured sample begin to wet after the test is started [15]. A shorter wetting time indicates the better ability of the fabric to manage liquid moisture. It is due to the fact that wetting determines the transport of liquid caused by capillary forces. This spontaneous flow of liquid resulting from the capillary forces is called wicking. Wicking can only occur when a liquid wets fibers assembled with capillary spaces between them. The MMT provides the values of wetting time for both surfaces of the measured specimen: top (inner, near to the human skin while wearing clothing) and bottom (outer side of clothing). 

The shortest wetting time of the top surface, 32.67 s, was stated for the KF3 knitted fabric—the green one. This was a cotton fabric with 3% of elastane, constructed in a pique stitch. The KF3 fabric was characterized by the smallest number of wales and rows in length units (Table 1). However, the KF3 fabric was characterized by the longest wetting time for the bottom surface—76.90 s. From the point of view of liquid sweat evaporation, this relation between the wetting time of the top and bottom surfaces is not satisfactory. In order to evaporate liquid sweat, it is advantageous to transport the liquid as quickly as possible to the bottom surface, which is closest to ambient air. The shortest wetting time for the bottom surface (6.48 s) was stated for the KF1 fabric, which is positive from the point of view of liquid moisture management. The surface becomes wet in a shorter time than for other investigated fabrics. The KF4 and KF5 fabrics are also characterized by short wetting times of the bottom surface: KF4—8.82 s and KF5—8.76 s. This is significantly shorter than the wetting time of the bottom surface for the KF2 (74.47 s) and KF3 (76.90 s) fabrics. However, in order to assess the fabrics from the point of view of liquid moisture transport, it is necessary to take into account also other aspects of liquid transport in the fabric, such as the absorption of the liquid and its spreading on both surfaces. 

The absorption rate (TAR, BAR) is the next parameter that is very important from the point of view of liquid transport in fibrous structures. The absorption rate is the average speed of liquid absorption for both surfaces of the measured specimen during the initial change in water content during a test [15]. The absorption of liquid by a fibrous material limits the movement of liquid caused by capillary forces. The greater the absorption is, the more limited the spreading the liquid on a surface. Moreover, a high absorption rate for the top (inner) surface (TAR) means that the liquid is trapped in the fibers of this inner surface and it is not drained to the outer surface and at the same time to the outside of the clothing. This is an unfavorable situation from the point of view of draining liquid sweat from the wearer’s skin to the outside. 

The highest value of the absorption rate for the top surface (351.06%/s) was stated for the KF3 fabric, followed by the KF1 (245.49%/s) and the KF2 (228.55%/s). The lowest absorption rate for the top surface occurred for the KF5 fabric (22.68%/s), followed by the KF4 fabric (77.66%/s). In the case of the absorption rate for the bottom surface, the highest absorption rate occurred for the KF4 fabric (77.92%/s) and the KF5 fabric (73.88%/s), with the lowest for the KF2 fabric (29.92%/s). In this case, the differences between the investigated fabric variants are not as high as in the case of the absorption rate for the top surface. 

The maximum wetted radius denotes the maximum radius of the sensor ring on which the liquid has been detected. The greater the maximum wetted radius is, the better the spreading of the liquid on the surface, and, at the same time, the better the conditions for liquid evaporation. In the context of liquid sweat evaporation from human skin to the environment, the maximum wetted radius on the bottom surface is more important than the maximum wetted radius on the top surface. A larger area of liquid spread on the bottom (outer) surface means better conditions for liquid evaporation from the space under the clothing into the ambient air. In the case of the fabrics being investigated, the greatest maximum wetted radius occurred for the KF5 fabric (10 mm), and the lowest for the KF2 and KF3 fabrics (2 mm). The highest value of the maximum wetted radius of the bottom surface for the KF5 fabric can be explained by the fact that it is composed of a cotton/polyester blend. The large share of polyester fibers (46%) is a key factor here. The polyester fibers are hydrophobic. They do not absorb water. At the same time, they do not retain liquid inside them. The liquid can travel from the upper to the bottom surface easily and quickly. Next, it can be spread on the bottom surface due to the capillary forces. 

The spreading speed is defined as the “accumulated rate of surface wetting from the centre of the specimen where the test solution is dropped to the maximum wetted radius” [15]. The higher the spreading speed is, especially on the bottom surface (SSB), the better the conditions for liquid evaporation. The greatest spreading speed of the bottom surface was stated for the KF5 fabric (2.05 mm/s), followed by the KF4 fabric (0.95 mm/s). The lowest spreading speed on the bottom surface occurred for the KF1 and KF2 fabrics, respectively, 0.31 mm/s and 0.32 mm/s. The results of the spreading speed on the bottom surface are in agreement with the results for the maximum wetted radius for the bottom surface. 

The accumulative one-way transport index R is a synthetic parameter. It is a measure of the difference between the areas of the liquid moisture content curves on the water content vs. time graphs obtained for the bottom and the top surfaces of a specimen with respect to time [24]. A high value of the parameter R means that the fabric ensures good accumulative one-way transport from the inner fabric side to the outer side, and, in the same way, it provides good sweat management for the clothing user. A fabric with a high accumulative one-way transport index keeps the skin of clothing wearer dry due to the transport of the liquid sweat from the inner surface to the outer surface, being away from the skin during clothing usage.

Very high values of the R parameter were stated for the fabrics KF5 (1021.32) and K4 (913.70). Negative values of the R parameter occurred for the fabrics KF3 (−370.50) and KF2 (R-59.64). The results of the R parameter are in agreement with the results of the BAR and MWRB parameters. 

The Overall Moisture Management Capability (OMMC) is calculated using the formula presented in AATCC Test Method 195-2017 [23]. The OMMC is based on the absorption rate for the bottom surface, spreading speed for the bottom surface and one-way transport capability with the appropriate weights. The value of the OMMC parameter is in the range of 0 to 1. A higher value of the OMMC parameter indicates the better capability of the fabric to manage liquid moisture. The values of the OMMC parameter (Table 3) are in full agreement with the R parameter. The best capability to manage liquid moisture was stated for the KF5 and KF4 fabrics, with the worst for the KF3 and KF2 fabrics. This was in line with our expectations taking into account the values of the parameters previously discussed. 

As was mentioned earlier, the best performance of the KF5 fabric may result from the fact that it contains 45% polyester fibers, which are hydrophobic and do not absorb water. Due to this fact, liquid can be spread on the fabric’s surfaces and then evaporated. 

Table 4 and Table 5 present the results from the MMT for the knitted fabrics stretched to 15%. 

Generally, it can be clearly seen that stretching changed the values of the parameters characterizing liquid moisture transport in the investigated knitted fabrics. It is also seen that the changes are different for different fabric variants. Summarizing the results for the stretched fabrics, it can be stated that the capability of the investigated fabrics to manage liquid moisture was improved in all cases. The most significant improvement was stated for the KF2 and KF3 fabrics. The KF5 fabric, which, in the relaxed (unstretched) state, was assessed as the best one among all investigated fabric variants, in the stretched form, showed the same level of the OMMC parameter. This denotes a similar capability of liquid moisture transport in the unstretched and stretched state. 

In the case of the KF2 fabric, stretching to 15% caused a significant reduction in the wetting time of the bottom surface, from 74.47 s to 6.012 s, and complete elimination of liquid absorption by the top surface. This means that the liquid dropped on the top surface travels directly through the pores in the fabric to the bottom surface and is spread on the bottom surface. For the KF2 fabric, after stretching, the maximum wetted radius on the bottom surface increased from 2 mm to 7 mm, whereas any spreading was observed on the top surface. The liquid transport performance of the KF3 fabric was also significantly improved. 

The MMT software classifies the investigated fabrics according to the test results into the following seven major types of fabrics:

- waterproof;
- water-repellent;
- slow-absorbing and slow-drying;
- fast-absorbing and slow-drying;
- fast-absorbing and quick-drying;
- water penetration fabric;
- moisture management fabric.

The classification of the investigated fabrics before and after stretching is presented in Table 6. 

Before stretching, the fabrics KF1, KF4 and KF5 were assessed as water penetration fabrics. This indicates a small spreading area and excellent one-way transport. The KF2 and KF3 fabrics, before stretching, were classified as waterproof fabrics. This indicates very slow absorption, slow spreading and no one-way transport, with no penetration [14]. After stretching, KF1, KF4 and KF5 were classified as the same type, water penetration fabrics, as before stretching. The KF2 and KF3 fabrics, previously assessed as waterproof, after stretching, were classified as water penetration fabrics. 

**Figure 4:**
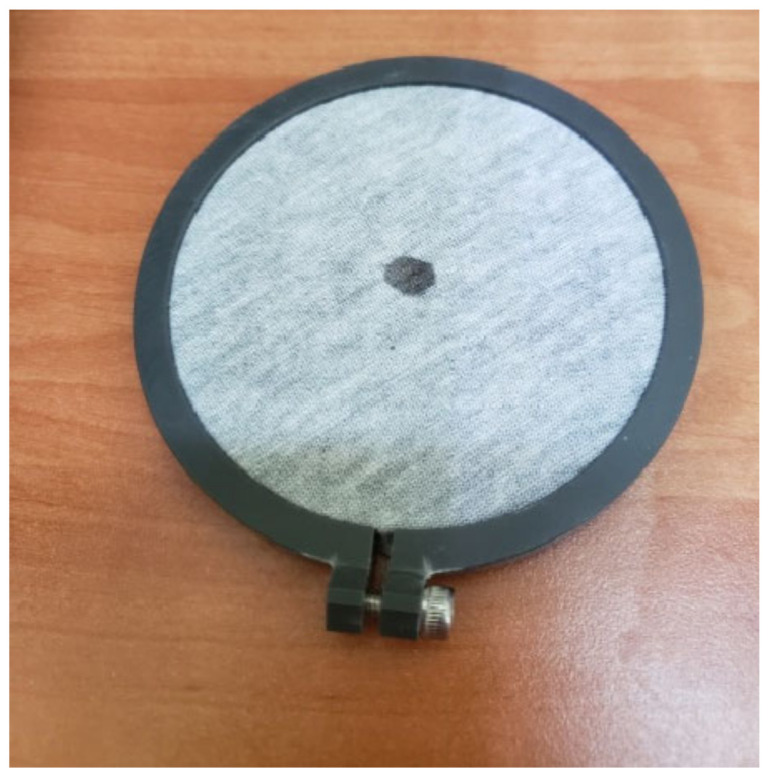
Liquid trace on the stretched KF4 fabric after test performance by means of the MMT.

It should be mentioned here that great variation in the results was observed for fabrics in the unstretched and stretched form. This phenomenon has been observed in our previous investigations too [20,21]. It can be explained by the fact that the testing liquid is dropped on the central region of the MMT sensor. Placing the samples on the bottom sensor is random. This means that different elements of the fabric structure can be positioned in the central point of the specimen measured, i.e., in the point where the liquid is applied. If a drop hits the open pores between the yarns, it can fall directly onto the bottom surface of the sample due to gravity. In such a case, the wetted radius is very small (Figure 4) and some amount of testing solution travels directly to the bottom sensor and remains on it. In such a situation, the amount of liquid absorbed and spread on the fabric surface is lower than that provided during the test. Unfortunately, it is impossible to determine the share (amount) of liquid in/on the measured fabric’s surface and the liquid on the bottom sensor’s surface. We can only observe the message on the PC screen indicating that the bottom sensor is wet and should be dried. 

**Figure 5:**
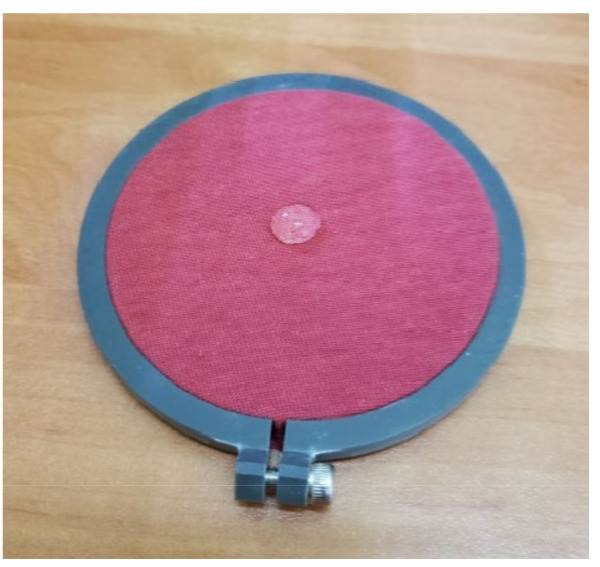
A drop of testing liquid on the fabric’s top surface after test performance by means of the MMT.

If, in turn, the drop hits the yarn, creating a loop, it can be transported through the capillaries between the yarns and fibers. It can also be partly absorbed by the fibers. In some situations, it was also observed that the fabrics were not wetted or wetting was negligible. In such a situation, the drops of water remained on the top surface of the fabric (Figure 5).

**Figure 6:**
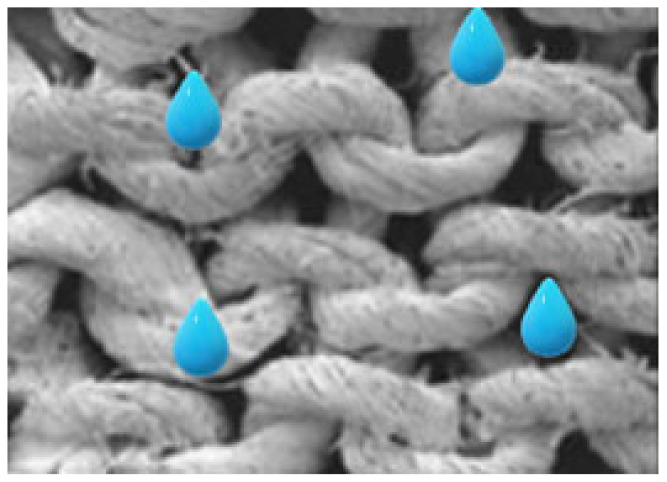
A schematic showing drops of testing liquid on different locations of stitch on the fabric surface.

During the measurements, all aforementioned cases were observed. This explains why different results were obtained for individual samples of a given fabric variant. In essence, the drop of the testing solution can fall randomly on different parts of the knitting stitch repeat. It can fall on the pores between yarns, on the head or between the heads of loops, as well on legs of the loop (Figure 6). In each case, the transport of liquid can occur in a different manner. 

However, there is no reason to consider any of the obtained results as outliers and to eliminate them. It should be taken into account that the material of the clothing adheres to the wearer’s skin over a large area when worn. This means that the sweat condensed on the human skin is also perceived by the clothing material in a different way, slightly differently in places with open pores, and slightly differently in places filled with yarns. In real conditions of clothing usage, the phenomenon of the removal of liquid sweat from the surface of the human skin is a result of the removal of sweat in individual places where the product comes into contact with the human skin. Due to this fact, it is stated that the calculation of the mean values of individual results, even with a large scatter of results, is the best solution to assess the capability of the fabrics to manage liquid sweat. This can reflect, in the best way, the real conditions of liquid sweat transport in clothing while wearing it. 

The results from the MMT were statistically analyzed using ANOVA. In the statistical analysis, the variants of the knitted fabrics—KF1, KF2, KF3, KF4, KF5—and the state—unstretched, stretched—were applied as independent variables, and the parameters from the MMT—WTT, WTB, TAR, BAR MWRT, MWRB, SST, SSB, R, OMMC—were applied as dependent variables. Each parameter from the MMT was analyzed separately. The statistical significance was assessed at the probability level of 0.95.

**Figure 7:**
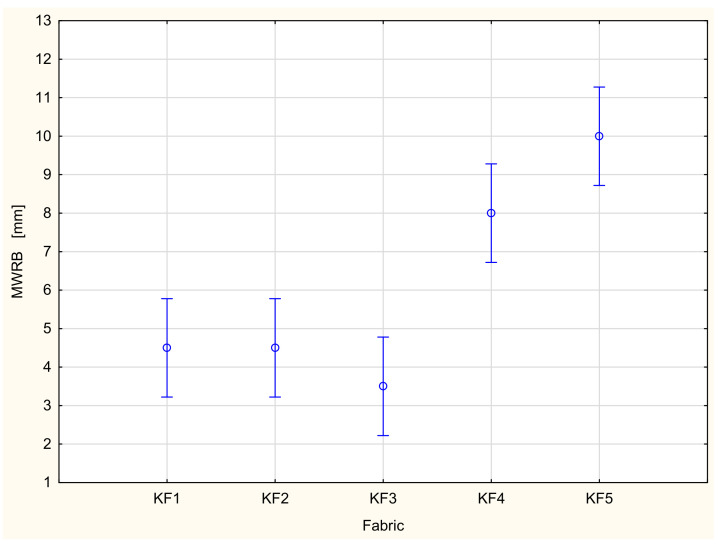
Influence of the fabric variant on the maximum wetted radius on the top surface.

**Figure 8:**
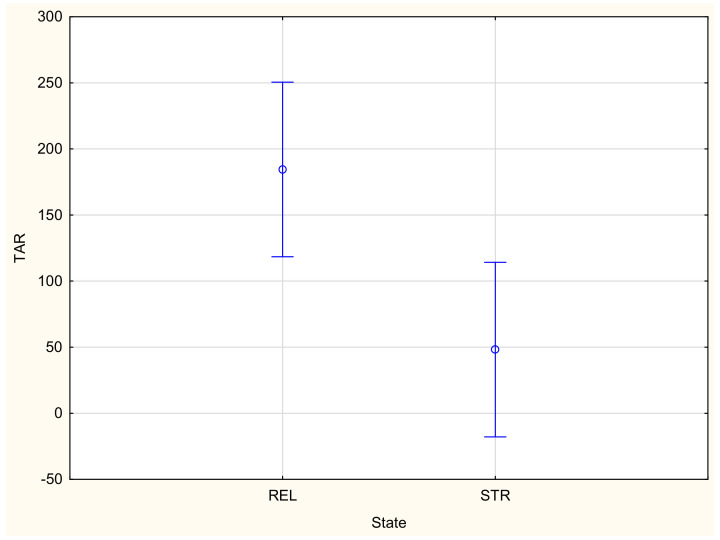
Influence of state on the absorption rate for the top surface: REL—relaxed (unstretched) state, STR—stretched state.

**Figure 9:**
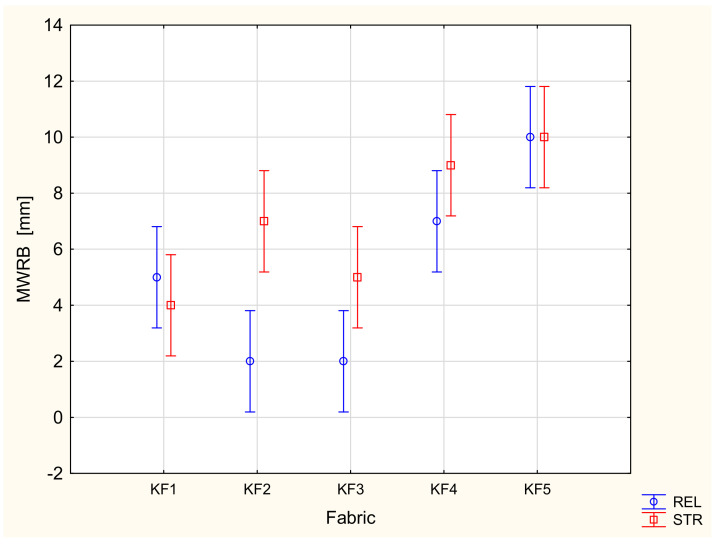
Influence of the fabric variant and state on the maximum wetted radius for the bottom surface: REL—relaxed (unstretched) state, STR—stretched state [23].

In the majority of cases, the results of the ANOVA confirmed that both main factors, the variant of the fabric and stretching, had a statistically significant influence on the parameters characterizing the liquid moisture transport through the knitted fabrics. This is in agreement with our previous studies [20,21]. The variant of the knitted fabrics significantly influenced the following parameters: TAR, SSB, MWRB (Figure 7), R and OMMC. The state (stretched/unstretched) had a statistically significant influence on WTT, WTB, TAR (Figure 8), SST, SSB, MWRT, MWRB, R and OMC. Only in the case of the BAR (absorption rate for the bottom surface), the influence of both independent variables was insignificant. The interaction between the independent variables was statistically significant only for two parameters: WTB and MWRB (Figure 9). 

## 4. Conclusions

The presented investigations of liquid moisture transport in knitted fabrics in unstretched and stretched form confirmed that the knitted fabrics applied in clothing worn close to human skin, such as T-shirts, are characterized by different abilities to transport liquid sweat produced by the human body. The MMT Stretch Fabric Fixture device makes it possible to prepare stretched samples for the measurement of liquid moisture transport by means of the MMT. The measurement of stretched knitted fabrics is very important because the underwear and other clothing worn close to the human skin is in a stretched form during usage. Their measurement in the unstretched form is not sufficient to assess the capability of the fabrics to ensure liquid moisture transport during usage. Stretching of the knitted fabrics changes their surface characteristics and the tightness of the structure, which influences the moisture transport. In the case of the investigated knitted fabrics, stretching the samples to 15% caused significant changes in all parameters characterizing the liquid moisture transport through them. Generally, the changes caused by stretching led to an improvement in their liquid moisture management ability. At the same as stretching, the levels of the changes in liquid transport characteristics were different for the different fabric variants being investigated. Preliminary investigations showed that it is difficult to reach more than 30% for standard clothing materials. Thick materials such as polar ones cannot be stretched using the MMT Stretch Fabric Fixture because they are too thick to be locked in the clamp. The question is how to stretch standard woven fabrics without elastomeric yarns. This will be a subject of our future investigations.

## Figures and Tables

**Table 1:** The basic structural parameters of the investigated knitted fabrics [23].

Knitted Fabric (Color)	Raw Material Composition, %	Stitch	Thickness, mm	Mass per Square Meter, gr/m^2^	Wale Density cm^−1^	Course Density cm^−1^
KF-1 red	100% cotton	Single jersey	0.47	161.09	16	22
KF-2 white	100% cotton	Rib stitch	0.61	138.59	12	17
KF-3 green	97% cotton, 3% elastane	Pique	1.27	198.44	10	12
KF-4 grey	95% cotton, 5% viscose	Single jersey	0.96	149.53	16	20
KF-5 black	54% cotton, 46% polyester	Single jersey	1.32	205.47	11	16

**Table 2:** Results from the MMT for the unstretched knitted fabrics [21].

Fabric	WTT [s]	WTB [s]	TAR [%/s]	BAR [%/s]	MWRT [mm]	MWRB [mm]
KF-1 red	55.52 (58.89)	6.48 (1.95)	245.49 (232.31)	50.85 8.64	3 (2.74)	5 (0)
KF-2 white	53.69 (60.55)	74.47 (62.34)	228.55 (256.16)	29.65 (40.74)	3 (2.74)	2 (2.74)
KF-3 green	32.67 (48.89)	76.90 (59.33)	351.06 (209.24)	65.0 (114.66)	4 (2.24)	2 (2.74)
KF-4 grey	63.86 (51.84)	8.82 (2.15)	74.66 (155.17)	77.92 (6.97)	3 (2.74)	7 (2.74)
KF-5 black	90.66 (46.16)	8.76 (2.56)	22.68 (44.47)	73.88 (30.44)	2 (2.74)	10 (0)

**Table 3:** Results from the MMT for the unstretched knitted fabrics, continuation [23].

Fabric	SST [mm/s]	SSB [mm/s]	R [−]	OMMC [−]
KF-1 red	0.24 (0.23)	0.80 (0.24)	424.31 (523.47)	0.41 (0.24)
KF-2 white	0.32 (0.30)	0.32 (0.44)	−59.64 (1093.38)	0.27 (0.37)
KF-3 green	0.38 (025)	0.31 (0.58)	−370.50 (880.32)	0.18 (0.29)
KF-4 grey	0.12 (0.12)	0.95 (0.68)	913.70 (248.48)	0.71 (0.05)
KF-5 black	0.08 (0.15)	2.05 (0.50)	1021.32 (193.55)	0.76 (0.10)

**Table 4:** Results from the MMT for the stretched knitted fabrics [23].

Fabric	WTT [s]	WTB [s]	TAR [%/s]	BAR [%/s]	MWRT [mm]	MWRB [mm]
KF-1 red	97.42 (50.48)	29.18 (50.78)	28.92 (64.67)	57.05 (32.07)	1 (2.23)	4 (2.23)
KF-2 white	120.00 (0)	6.12 (0.93)	0.00 (0)	67.06 (67.06)	0 (0)	7 (2.74)
KF-3 green	79.21 (55.88)	8.24 (5.55)	196.80 (269.49)	83.07 (34.26)	2 (2.74)	5 (0)
KF-4 grey	120.00 (0)	8.09 (2.23)	0.00 (0)	145.16 (144.95)	0 (0)	9 (2.24)
KF-5 black	76.68 (59.40)	13.82 (19.40)	15.04 (26.15)	172.57 (220.82)	2 (2.74)	10 (0)

**Table 5:** Results from the MMT for the stretched knitted fabrics, continuation [23].

Fabric	SST [mm/s]	SSB [mm/s]	R [-]	OMMC [-]
KF-1 red	0.47 (0.31)	0.60 (0.34)	690.39 (935.82)	0.54 (0.30)
KF-2 white	0.00 (0.15)	1.61 (1.13)	1091.92 (23.51)	0.72 (0.08)
KF-3 green	0.11 (0.15)	0.73 (0.26)	644.93 (653.74)	0.50 (0.25)
KF-4 grey	0.00 (0)	2.09 (0.84)	1177.25 (68.21)	0.80 (0.07)
KF-5 black	0.20 (0.30)	2.29 (0.96)	963.93 (388.60)	0.77 (0.11)

**Table 6:** Classification of the investigated fabrics into types.

Fabric	Before Stretching	After Stretching
KF-1 red	Water penetration fabric	Water penetration fabric
KF-2 white	Waterproof fabric	Water penetration fabric
KF-3 green	Waterproof fabric	Water penetration fabric
KF-4 grey	Water penetration fabric	Water penetration fabric
KF-5 black	Water penetration fabric	Water penetration fabric

## Data Availability

The data presented in this study are available on request from the corresponding author.

## References

[1] Eryuruk S.H., Koncar V., Kalaoglu F., Gidik H., Tao X. (2018). Thermal comfort properties of firefighters’ clothing with underwear. IOP Conference Series: Materials Science and Engineering.

[2] Mansor A., Ghani S.A., Yahya M.F. (2016). Knitted Fabric Parameters in Relation to Comfort Properties. Am. J. Mater. Sci..

[3] Bajzik V., Hes L., Dolezal I. (2016). Changes in thermal comfort properties of sportswear and underwear due to their wetting. Indian J. Fibre Text. Res..

[4] Elrys S.M., El-Habiby F.F., Eldeeb A.S., El-Hossiny A.M., Abd Elkhalek R. (2022). Comfort properties of knitted fabrics produced from dual-core and tri-core spun yarns. Text. Res. J..

[5] Gorji M., Bagherzadeh R. (2016). Moisture Management behaviors of high wicking fabrics composed of Profiled Fiber. Indian J. Fibre Text. Res..

[6] Choudhary A.K., Ramratan (2020). The Influence of Yarn and Knit Structure on Moisture Management Properties of Sportswear. Fabric. J. Inst. Eng. Ser. E.

[7] Manshahia M., Das A. (2014). Moisture management of high active sportswear. Fiber. Polym..

[8] Ziemele I., Šroma I., Kakarāne A. (2018). Comfort in Sportswear. Key Eng. Mater..

[9] Dolez P.I., Marsha S., McQueen R.H. (2022). Fibers and Textiles for Personal Protective Equipment: Review of Recent Progress and Perspectives on Future Developments. Textiles.

[10] Khalil A., Tesinova P., Aboalasaad A. (2021). Thermal comfort properties of cotton/spandex single jersey knitted fabric. Ind. Text..

[11] Khalil A., Fouda A., Těšinová P., Eldeeb A.S. (2021). Comprehensive assessment of the properties of cotton single jersey knitted fabrics predicted from diferent lycra states. AUTEX Res. J..

[12] Mishra R., Jamshaid H., Yosfani S.H.S., Hussain U., Nadeem M., Petru M., Tichy M., Muller M. (2021). Thermo physiological comfort of single jersey knitted fabric derivatives. Fash. Text..

[13] Barnes K.A., Anderson M.L., Stofan J.R., Dalrymple K.J., Reimel A.J., Roberts T.J., Randell R.K., Ungaro C.T., Baker L.B. (2019). Normative data for sweating rate, sweat sodium concentration, and sweat sodium loss in athletes: An update and analysis by sport. J. Sports Sci..

[14] Instruction Manual SDL Atlas M 290 MMT Manual. Rev. (01/17) 1.2 SDL Atlas 2017.

[15] (2012). Liquid Moisture Management Properties of Textile Fabrics.

[16] Matusiak M. (2019). Moisture Management Properties of Seersucker Woven Fabrics of Different Structure. Fibres Text. East. Eur..

[17] Zou C., Lao L., Chen Q., Fan J., Shou D. (2021). Nature-inspired moisture management fabric for unidirectional liquid transport and surface repellence and resistance. Energy Build..

[18] Mayur B., Mrinal C., Saptarshi M.R., Adivarekar R.V. (2018). Moisture Management Properties of Textiles and Its Evaluation. Curr. Trends Fash. Technol. Text. Eng..

[19] (2020). MMT Stretch Fabric Fixture, Instruction Manual.

[20] Matusiak M., Froschauer S. Influence of stretching on liquid moisture transport in knitted fabrics. Proceedings of the 21st World Textile Online Conference AUTEX 2022.

[21] Matusiak M., Sukhbat O. (2022). Liquid Moisture Transport in Knitted Fabrics in Relaxed and Stretched State. Commun. Dev. Assem. Text. Prod..

[22] Suganthi T., Senthilkumar P. (2018). Moisture-management properties of bi-layer knitted fabrics for sportswear. J. Ind. Text..

[23] Chen Q., Shou D., Zheng R., Fan J., Wan X., Fu B., Ma P. (2020). The Moisture Management and Drying Properties of Weft Knitted Plating Fabrics. Fibers Polym..

[24] Kumar T.S., Kumar M.R., Prakash C., Kumar B.S. (2022). Study on Moisture Management Properties of Plated Interlock Knitted Fabrics. J. Nat. Fibers.

